# Clinical significance of redundant nerve roots in patients with lumbar spinal stenosis undergoing oblique lumbar interbody fusion combined with percutaneous internal fixation

**DOI:** 10.1186/s13018-023-04449-2

**Published:** 2023-12-13

**Authors:** Hongzhou Sun, Shouliang Xiong, Yu Zhang, Quanlai Zhao, Zhongxuan Wu, Liang Xiao

**Affiliations:** 1 Department of Spine Surgery, Yijishan Hospital, The First Affliated Hospital of Wannan Medical College, Wuhu, China; 2 Department of Joint Surgery, Yijishan Hospital, The First Affliated Hospital of Wannan Medical College, Wuhu, China; 3 Spine Research Center of Wannan Medical College, No. 22 Wenchang West Road, Wuhu, 241001 China

**Keywords:** Redundant nerve roots, Lumbar spinal stenosis, Oblique lumbar interbody fusion, Clinical efficacy, Percutaneous internal fixation

## Abstract

**Background:**

While there have been previous studies on the surgical efficacy of patients with redundant nerve roots (RNRs), a persistent issue is that some patients continue to experience redundancy even after surgery. Furthermore, the clinical significance of RNRs remains unclear. Notably, there is a lack of research regarding RNRs within the context of oblique lumbar interbody fusion (OLIF) combined with percutaneous internal fixation. Therefore, the primary objective of this study is to investigate the correlation between RNRs and clinical outcomes following OLIF combined with percutaneous internal fixation.

**Methods:**

Eighty-seven patients diagnosed with lumbar spinal stenosis (LSS) who underwent single-segment OLIF combined with percutaneous internal fixation were categorized into three groups. Group 1 comprised patients with positive RNRs both before and after the operation. Group 2 included patients with positive RNRs preoperatively but negative RNRs postoperatively. Group 3 consisted of patients with consistently negative RNRs before and after the operation. Comprehensive patient data were collected, including operation time, intraoperative blood loss, and any recorded complications. Radiographic parameters, both pre- and post-operative, were assessed, encompassing the number of stenosis segments, disc height (DH), lumbar lordotic angle, dural sac cross-sectional area, and the placement of the fusion cage. Furthermore, the Visual Analogue Scale was applied to gauge back and leg pain, while the Oswestry Disability Index was employed to appraise daily living activities. A comparative analysis was carried out among the three patient groups.

**Results:**

In this study, all 87 LSS patients successfully underwent surgery. Among them, 35 patients (40.2%) showed preoperative MRI assessment indicating positive RNRs. In the postoperative MRI assessment, 14 of these patients maintained positive RNRs status, and they were grouped into Group 1. The remaining 21 patients saw a transition to negative RNRs status and were included in Group 2. Among the 52 patients who had preoperative MRI assessments showing negative RNRs, their postoperative RNRs status remained negative, forming Group 3. All patients received follow-up, which ranged from 8 to 18 months, and no complications occurred during this period. In this study, the postoperative efficacy and parameters such as DH and Dural Sac CSA significantly improved compared to preoperative values for all 87 patients. Patients with preoperative RNRs had more stenosis segments, smaller dural sac CSA, and more severe symptoms. In all three groups, postoperative efficacy scores significantly improved compared to preoperative scores. Group 2 patients had their fusion cages placed more in the middle, while Group 1 patients had their fusion cages more anteriorly located. Group 2 patients exhibited greater recovery in dural sac CSA postoperatively compared to Group 1 patients. Additionally, Group 2 patients had better ODI efficacy scores compared to Group 1 patients.

**Conclusions:**

Irrespective of the presence or absence of RNRs, patients experienced improvement after undergoing OLIF combined with percutaneous internal fixation. Preoperative RNRs appear to be linked to multi-segmental lumbar spinal stenosis, a reduction in dural sac CSA, and symptom severity. Patients with negative postoperative RNRs demonstrated better treatment efficacy. Furthermore, the placement of the fusion cage appears to have a significant impact on postoperative efficacy and RNRs outcomes.

## Background

Lumbar spinal stenosis (LSS) is the most prevalent degenerative spinal condition among the elderly. It is characterized by neurogenic claudication, lower back pain, and sensory disturbances in the lower extremities, often necessitating lumbar surgery [1, 2]. When compared to conservative treatment, decompression surgery offers notable advantages in the management of lumbar spinal stenosis [3–6]. Lumbar interbody fusion (LIF) is a mature surgical method for the treatment of LSS [7], LIF can be performed through a variety of different approaches, each of which has its unique advantages and limitations. With the continuous development of surgical techniques in the direction of precision and minimally invasive, a variety of minimally invasive interbody fusions have emerged. Among them, the oblique approach of LIF was first proposed in 2012, and Oblique lumbar interbody fusion (OLIF) has been more and more widely used in clinical practice in recent years due to its excellent surgical results [8].

Verbiest [9] first reported the clinical symptoms of LSS in 1954, and described the tortuosity of the cauda equina by myelography. In 1968, Cressman and Pawl [10] first introduced the real descriptive term Redundant nerve roots ( RNRs), which refers to spinal stenosis caused by increased epidural pressure, which in turn causes the cauda equina to be entangled, meandering, and tortuous. It is reported that the incidence of RNRs in LSS patients is 33.8% to 42.3% [11]. LSS is considered to be one of the main causes of the development of this symptom [12].

Since the first introduction of RNRs, some studies have reported the treatment and prognosis of patients with RNRs [12–16]. But an unsolved problem is that some patients remain redundant after surgery. In addition, the clinical significance of RNRs is still unclear, and RNRs have not been studied in the context of OLIF combined with percutaneous internal fixation. Therefore, the main purpose of this study is to investigate the relationship between RNRs and clinical outcomes after OLIF combined with percutaneous internal fixation.

## Patients and methods

### General information

We conducted a retrospective analysis to study the clinical data of LSS patients who underwent OLIF combined with percutaneous internal fixation in our hospital from June 2019 to June 2022. All surgeries were performed by the same medical team. Finally, we included 87 patients who met the above criteria, including 30 males and 57 females, aged 44–82 (63.55 ± 9.99) years. All patients were followed up for 8–18 (11.04 ± 3.61) months, and no complications were found during the follow-up period. Inclusion criteria: (1) patients with neurogenic intermittent claudication; (2) There was central lumbar spinal stenosis; (3) The results of lumbar MRI examination were complete and clear, and the surgical segment was limited to a single level. (4) There was no obvious instability of the lumbar spine; (5) All patients underwent the same surgical method, namely OLIF combined with percutaneous internal fixation. Exclusion criteria: (1) patients with spinal trauma, lumbar infection, tumor or nerve injury; (2) Patients with a history of lumbar surgery; (3) Spinal canal stenosis caused by lumbar spondylolysis. This study was approved by the ethics committee of our hospital. All patients signed the informed consent of this study. (4) Patients with lumbar disc free and prolapse. Details are shown in Table 1.

**Table 1:** Patient demographic data

Parameter	Value
Sex ratio (M: F)	30/57
Age, mean (range), years	63.55 ± 9.99 (44–82)
Symptom duration, (mean), months	41.55 ± 68.94
Height, (mean), cm	163.67 ± 6.60
Weight, (mean), kg	65.25 ± 8.43
BMI, (mean), kg/m^2^	24.34 ± 2.67
Number of stenosis segments	1.38 ± 0.49
Operation time, (mean), hours	2.10 ± 1.02
Blood loss, (mean), ml	79.31 ± 39.85

BMI, Body mass index

### Surgical procedures

All patients underwent the same surgical procedure. The specific steps are as follows: Before the operation, the patient 's blood glucose and blood pressure were controlled to ensure the stability of cardiopulmonary function. After successful implementation of general anesthesia, the patient was placed in the right lateral position, and the waist and iliac transition area were placed on the waist bridge of the operating table, and the waist bridge was elevated. At the same time, an armpit pad is used to protect the armpit area, flex the hip and knee joints, and a folding pad is used to isolate and protect the lower limbs. The patient 's position was fixed by a cloth band, and the responsible segment of the operation was determined by C-arm X-ray fluoroscopy, and marked on the body surface. A 3-5 cm incision was made about 3 cm forward from the midpoint of the target intervertebral disc, and then blunt separation was performed along the direction of the abdominal muscle fibers into the retroperitoneal space. At the leading edge of the psoas major, the index finger was used to separate the peritoneal tissue to expose the responsible intervertebral disc. Then, the probe was inserted into the intervertebral space, and the expander kit was gradually implanted to distract the abdominal muscle fibers layer by layer to expose the responsible intervertebral disc. Then, the nucleus pulposus tissue of the intervertebral disc was removed and the cartilage endplate was scraped. Finally, the lateral interbody fusion cage filled with bone graft material was placed in the target intervertebral space, and X-ray fluoroscopy was used to confirm whether the placement position was well reset. Subsequently, the incision was sutured layer by layer and pressure dressing was performed. Subsequently, the patient was changed to the prone position, and the internal fixation rod was placed percutaneously on both sides of the surgical segment under fluoroscopy guidance. After surgery, all patients received conventional antibiotics to prevent infection, and bed rest for 3–7 days. Two weeks later, back muscle function exercise was started, and strenuous waist or heavy physical activity was avoided in the first three months after surgery.

### Evaluation of surgical outcomes and measurements of Radiographic parameters

**Fig. 1:**
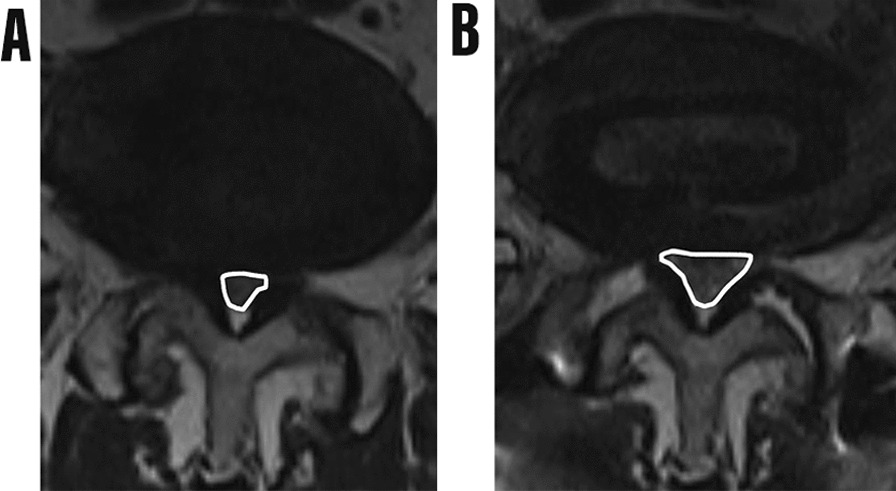
Preoperative and postoperative dural sac CSA was evaluated by MRI imaging. **A** Preoperative dural sac CSA. **B** Postoperative dural sac CSA

The general data of all patients were completely recorded, including operation time, intraoperative blood loss and any possible complications. Magnetic resonance imaging (MRI) and X-ray plain film were used for imaging studies. Before and 7 days after surgery, all patients underwent MRI scans, including high-resolution T2 axial and sagittal sequences. During the scanning, the patient was kept in supine position. The scanning range included L2 / 3, L3 / 4, L4 / 5 and L5 / S1 segments, and each segment was scanned by 3 layers. We used the PACS imaging system to measure the cross-sectional area (CSA) (cm^2^) of the dural sac at the maximum stenosis level of the intervertebral disc. The dural sac CSA was determined by the dural sac contour area on the T2-weighted axial MRI image (Fig. 1) [17].

**Fig. 2:**
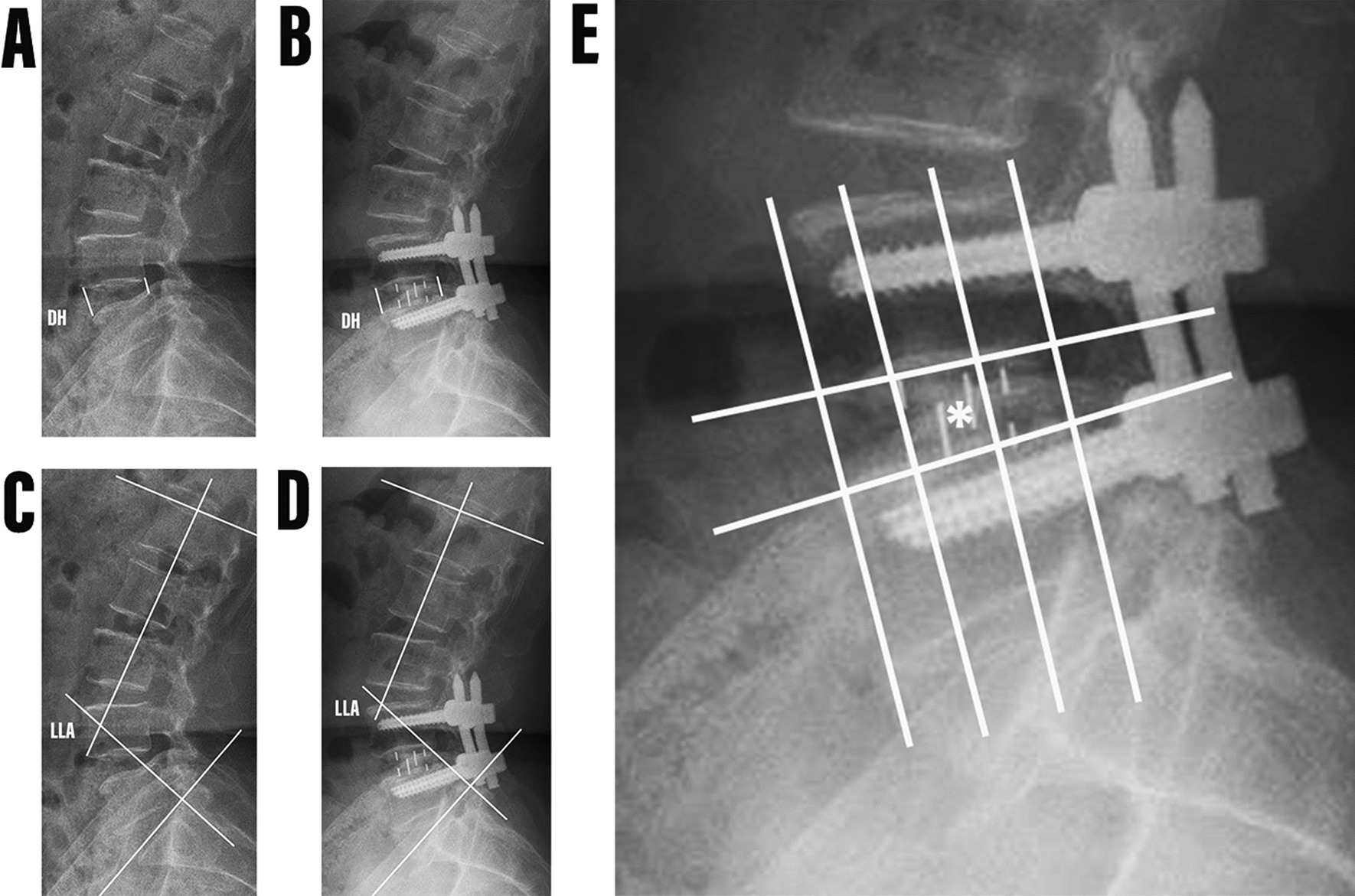
Measurement of Disc height (DH) and lumbar lordosis angle (LLA) by lateral X-ray before and after operation. **A** Preoperative DH. **B** Postoperative DH. **C** preoperative LLA. **D** Postoperative LLA. **E** According to the position of the midpoint of the cage (*) in the intervertebral space, the cage position is considered to have an anterior, middle and posterior position

In addition, we performed anteroposterior and lateral X-ray examinations of all patients before and at the last follow-up after surgery. On lateral radiographs, we measured disc height (DH) (cm) and lumbar lordotic angle (LLA) (°) (Fig. 2). DH was defined as the average value of the anterior and posterior height of the intervertebral space of the surgical segment, and LLA was defined as the Cobb angle between the upper endplate of L1 and the upper endplate of the sacrum.

**Fig. 3:**
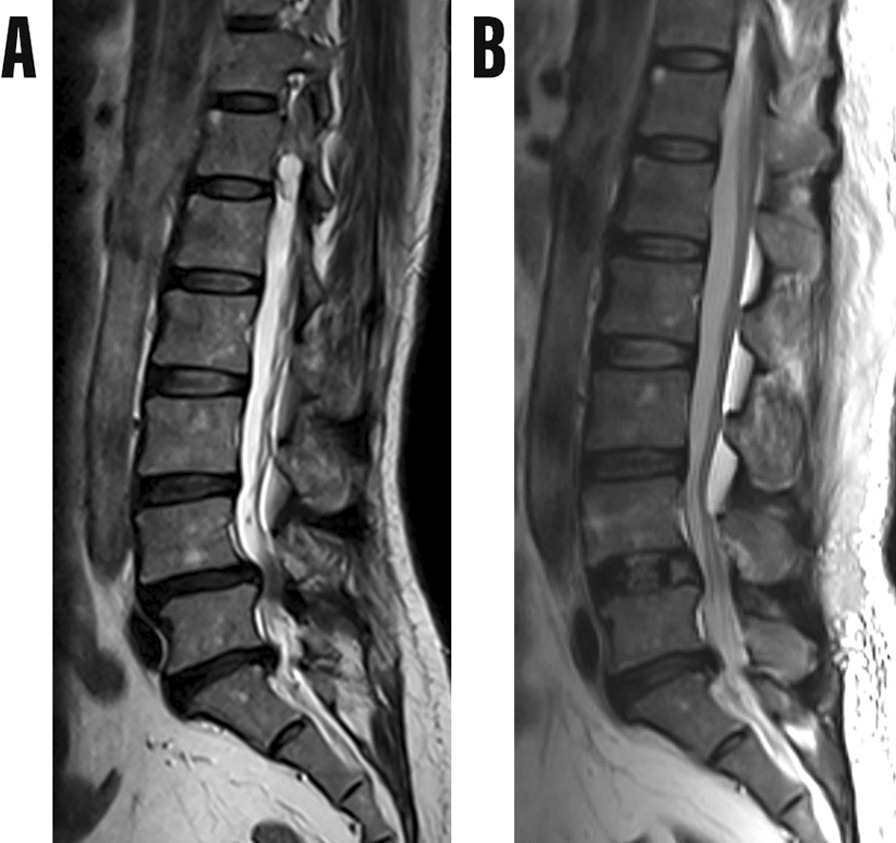
RNRs changed from positive to negative. **A** Preoperative RNRs positive. **B** Postoperative RNRs negative

**Fig. 4:**
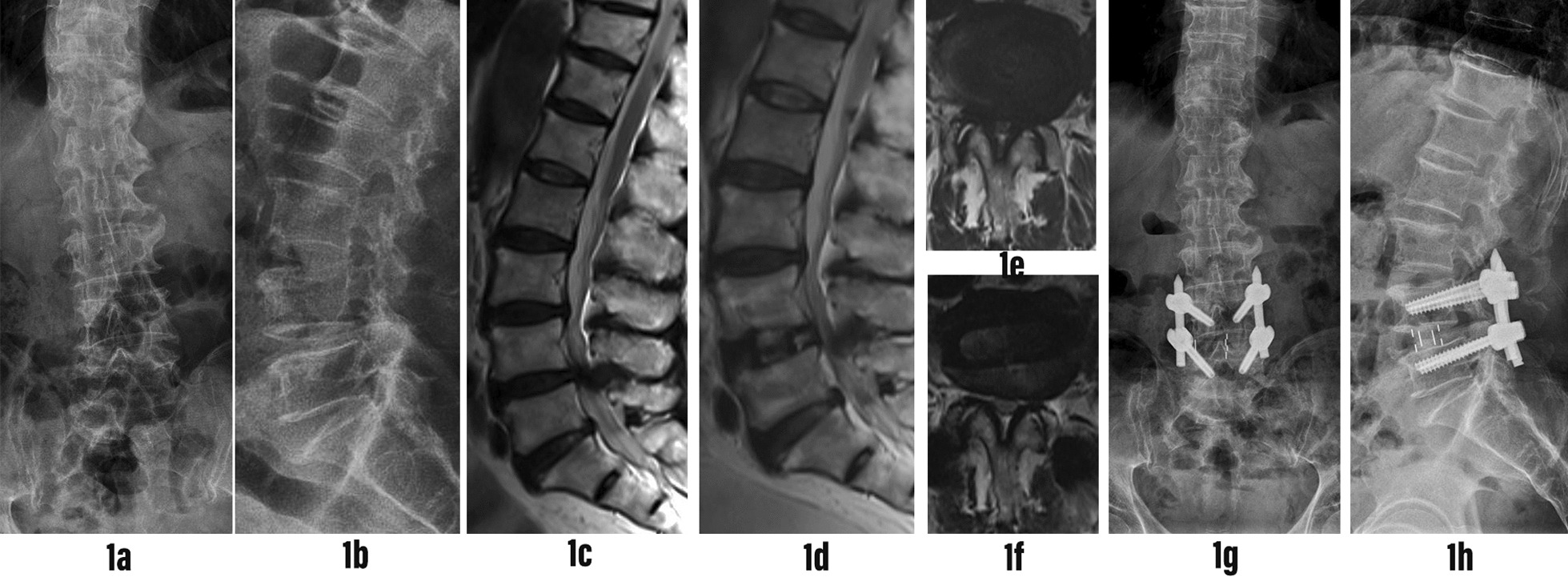
Typical case 1: A 70-year-old male patient presented with numbness of both lower limbs for more than 5 years, aggravated with left lower limb weakness for more than 1 month.Diagnosis of L4-5 spinal stenosis. L4-5 single segment OLIF combined with posterior L4-5 percutaneous internal fixation was performed. **1a, 1b** Anteroposterior and lateral X-ray films of lumbar spine before operation showed lumbar degeneration. **1c** Preoperative sagittal T2 MRI of the lumbar spine showed spinal stenosis in L4-5 segment, and RNRs above the stenosis plane. **1d** Postoperative sagittal T2 MRI of the lumbar spine showed that RNRs still existed above the L4-5 surgical segment. **1e, 1f** Axial T2 MRI of lumbar spine before and after operation showed that the dural sac CSA increased significantly after OLIF indirect decompression. **1g, 1h** At the last follow-up, the X-ray film of the lumbar spine showed that the L4-5 pedicle screw was fixed and the fusion cage was placed in a good position

**Fig. 5:**
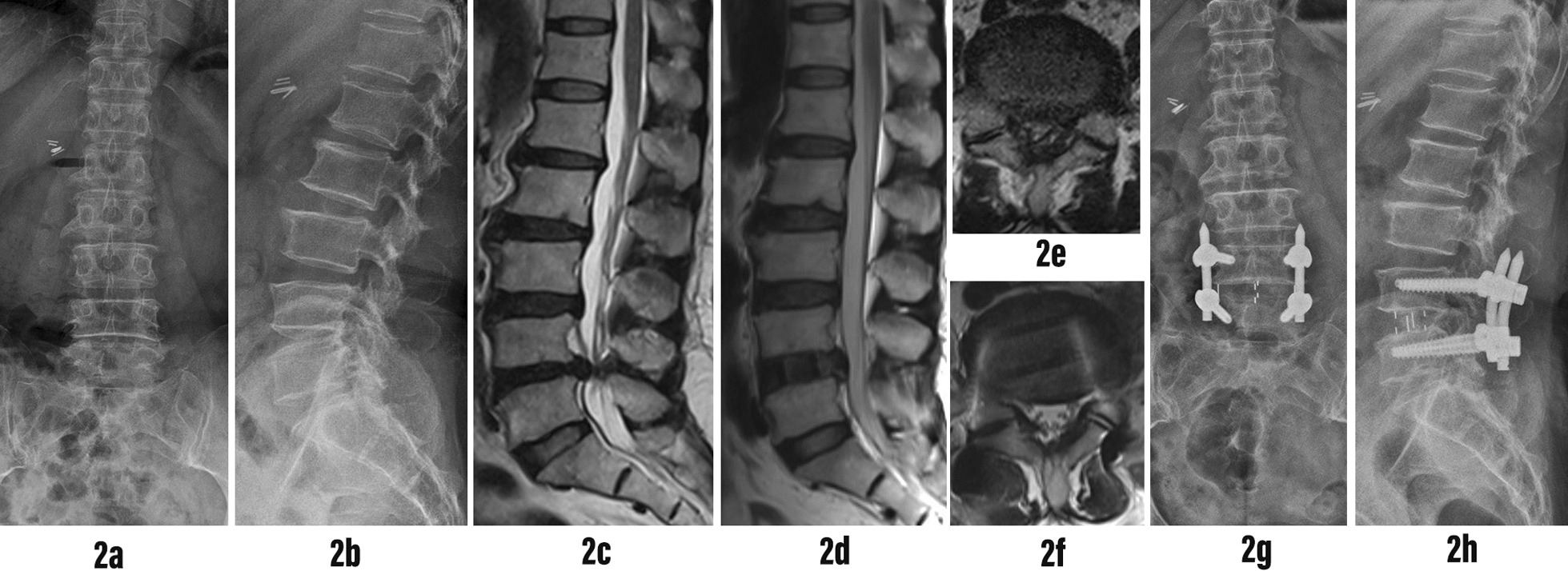
Typical case 2: A 76-year-old female presented with low back discomfort and right lower limb numbness for more than 2 years, which aggravated for 1 week. Diagnosis of L4-5 spinal stenosis. L4-5 single segment OLIF combined with posterior L4-5 percutaneous internal fixation was performed. **2a, 2b** Anteroposterior and lateral X-ray films of lumbar spine before operation showed lumbar degeneration. **2c** Preoperative sagittal T2 MRI of the lumbar spine showed severe spinal stenosis at L4-5 segment. RNRs was seen above the stenosis plane. **2d** Postoperative sagittal T2 MRI of the lumbar spine showed that the RNRs above the L4-5 surgical segment disappeared. **2e, 2f** Axial T2 MRI of lumbar spine before and after operation showed that the dural sac CSA increased significantly after OLIF indirect decompression. **2g, 2h** At the last follow-up, the X-ray film of the lumbar spine showed that the L4-5 pedicle screw was fixed, the cage was placed in the middle and the position was good, and the height of the L4-5 intervertebral space was recovered well

**Fig. 6:**
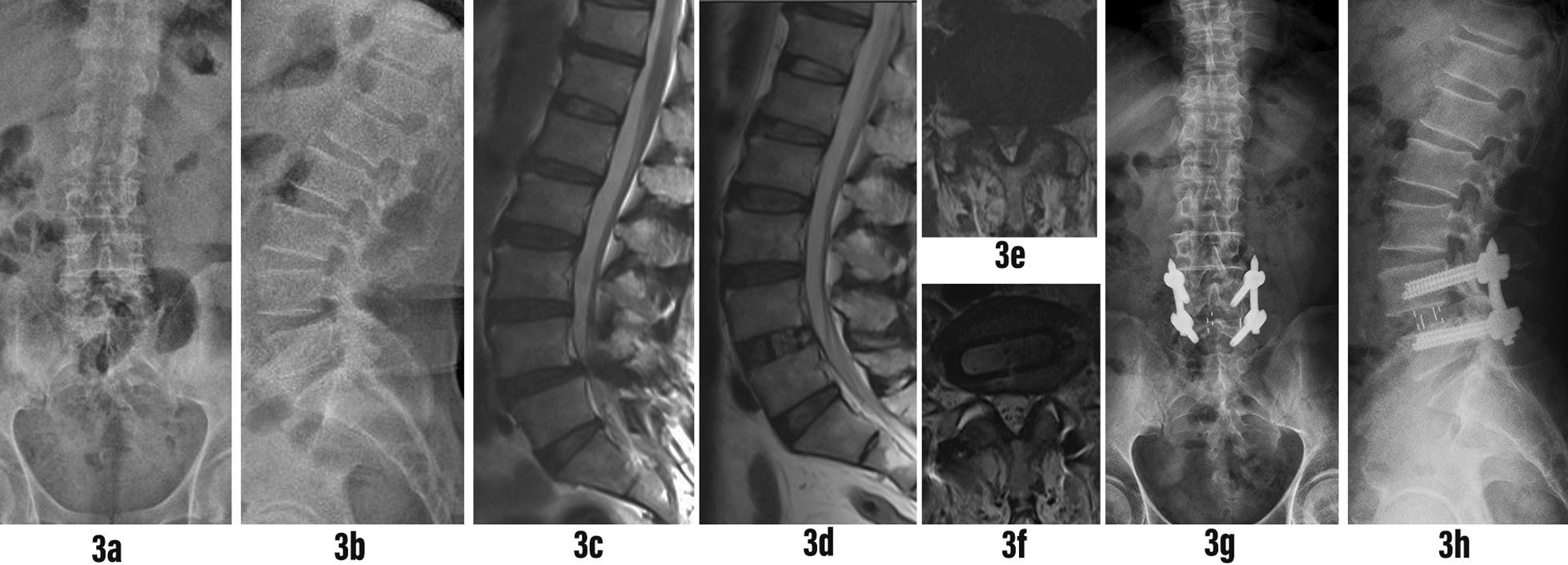
Typical case 3: A 74-year-old male presented with numbness in both lower limbs for more than 1 year, aggravated for 1 week. Diagnosis of L4-5 spinal stenosis with L4 vertebral 1 degree spondylolisthesis. L4-5 single segment OLIF combined with posterior L4-5 percutaneous internal fixation was performed. **3a, 3b** Anteroposterior and lateral X-ray films of the lumbar spine before operation showed lumbar degeneration and I ° spondylolisthesis of L4 vertebral body. **3c** Preoperative sagittal T2 MRI of the lumbar spine showed L4-5 spinal stenosis, no RNRs above the stenosis plane. **3d** Postoperative sagittal T2 MRI of the lumbar spine showed no RNRs above the L4-5 surgical segment. **3e, 3f** Axial T2 MRI of lumbar spine before and after operation showed that the dural sac CSA increased significantly after OLIF indirect decompression. **3g, 3h** At the last follow-up, the X-ray film of the lumbar spine showed that the L4-5 pedicle screw was fixed, good reduction of L4-5 spondylolisthesis, the fusion cage was placed in a good position, and the height of the L4-5 intervertebral space was well restored

According to the difference between the midpoint of the fusion cage and the intervertebral space, we divided the position of the fusion cage into three different positions: anterior, middle and posterior [18] (Fig. 2). In actual measurements, all measurements, including length, angle and area, were independently measured by three experienced spine surgeons using the PACS imaging system. The three doctors analyzed the sagittal T2 images of 92 patients, respectively. According to the existence of RNRs defined as the winding, winding and tortuosity of the cauda equina nerve in the spinal canal on the sagittal T2 image described by Cressman et al. [10] (Fig. 3). 87 patients diagnosed with LSS who underwent single-segment OLIF combined with percutaneous internal fixation were categorized into three groups. Group 1 comprised patients with positive RNRs both before and after the operation (Fig. 4). Group 2 included patients with positive RNRs preoperatively but negative RNRs postoperatively (Fig. 5). Group 3 consisted of patients with consistently negative RNRs before and after the operation (Fig. 6). Furthermore, the Visual Analogue Scale (VAS) [19]was applied to gauge back and leg pain, while the Oswestry Disability Index (ODI) [20]was employed to appraise daily living activities.

### Statistical analyses

SPSS 18.0 (SPSS Inc., Chicago, IL, USA) was used for statistical analyses. The measurement data were expressed as mean ± standard deviation according to the normal distribution. The difference between the measurement data before and after surgery (DH, LLA, dural sac CSA, VAS, ODI score) was compared by paired t test. The difference between the two groups of measurement data (operation time, intraoperative blood loss, DH, LLA, dural sac CSA) was analyzed by independent sample t test. The difference between the three groups of measurement data (age, course of disease, Body mass index (BMI), number of narrow segments, DH, LLA, dural sac CSA, VAS, ODI score) was analyzed by single factor analysis of variance. The difference between the count data (gender, fusion position) was analyzed by X^2^ test, and the difference was statistically significant with *P* < 0.05.

## Results

All patients observed varying degrees of improvement in symptoms after surgery. All patients were successfully discharged from hospital. The patients were followed up for 8–18 (11.04 ± 3.61) months. No complications occurred during the follow-up period. The VAS score of low back pain decreased from 4.85 ± 0.96 to 3.16 ± 0.82 (last follow-up), the VAS score of leg pain decreased from 4.43 ± 0.95 to 3.02 ± 0.90 (last follow-up), the ODI score decreased from 50.69 ± 5.55% to 41.36 ± 6.13% (last follow-up), DH increased from 1.02 ± 0.23 cm to 1.52 ± 0.23 cm, and dural sac CSA increased from 0.60 ± 0.26 cm^2^ to 0.98 ± 0.40 cm^2^. The change of LLA was not statistically significant. Details are shown in Table 2.

**Table 2:** Comparative analysis of pre- and post-operative data in 87 patients

	Preoperative	Postoperative	Paired t value	*P*
Number	87	87		
DH (cm)	1.02 ± 0.23	1.52 ± 0.23	19.933	0.000
LLA (°)	44.23 ± 13.36	44.03 ± 12.48	0.191	0.849
CSA (cm^2^)	0.60 ± 0.26	0.98 ± 0.40	11.271	0.000
low back pain VAS (score)	4.85 ± 0.96	3.16 ± 0.82	20.148	0.000
leg pain VAS (score)	4.43 ± 0.95	3.02 ± 0.90	12.044	0.000
ODI (%)	50.69 ± 5.55	41.36 ± 6.13	25.589	0.000

DH, Disc height; LLA, Lumbar lordotic angle; CSA, cross-sectional area; VAS, visual analogue scale; ODI, Oswestry disability index

In this study, all 87 LSS patients successfully underwent surgery. Among them, 35 patients (40.2%) showed preoperative MRI assessment indicating positive RNRs. In the postoperative MRI assessment, 14 of these patients maintained positive RNRs status, and they were grouped into Group 1. The remaining 21 patients saw a transition to negative RNRs status and were included in Group 2. Among the 52 patients who had preoperative MRI assessments showing negative RNRs, their postoperative RNRs status remained negative, forming Group 3. In order to understand these three groups of patients more comprehensively, we compared the preoperative clinical data of the three groups of patients. The results showed that there were no significant differences in Age, Sex, Symptom duration, BMI among these patients. However, the number of stenosis segments in group 1 and group 2 was more than that in group 3, and the difference was significant, as shown in Table 3. Compared with the preoperative imaging and scores of the three groups of patients, the preoperative symptoms of RNRs patients were more serious, and the preoperative dural sac area was smaller, the difference was statistically significant, as shown in Table 4. The postoperative curative effect scores of the three groups were significantly improved compared with those before operation, and the difference was statistically significant, as shown in Tables 5,6,7.

**Table 3:** Comparative analysis of general patient data among three groups

	Group 1	Group 2	Group 3	t/X^2^	*P*
Number	14	21	52		
Age (years)	65.50 ± 9.22	65.81 ± 11.29	62.11 ± 9.55	1.352	0.264
Sex ratio (M: F)	6:8	8:13	16:36	0.873	0.646
Symptom duration (months)	44.21 ± 44.80	47.33 ± 78.95	38.50 ± 70.95	0.132	0.876
Height (cm)	164.28 ± 5.94	165.19 ± 5.07	162.88 ± 7.26	0.985	0.378
Weight (kg)	65.21 ± 7.85	66.02 ± 8.18	64.94 ± 8.80	0.121	0.886
BMI (kg/m^2^)	24.10 ± 2.13	24.19 ± 2.81	24.47 ± 2.79	0.151	0.860
Number of stenosis segments	1.86 ± 0.36	1.62 ± 0.50	1.15 ± 0.36^#^	22.028	0.000

^#^Indicates that the difference between Group 3 and Group 2, Group 3 and group 1 is statistically significant

BMI, Body mass index

**Table 4:** Comparative analysis of radiographic and scoring among three patient groups

	Group 1	Group 2	Group 3	t/X^2^	*P*
Number	14	21	52		
Preoperative DH (cm)	1.08 ± 0.17	1.11 ± 0.22	0.97 ± 0.23	3.967	0.023
Preoperative LLA (°)	39.81 ± 10.44	45.93 ± 14.78	44.73 ± 13.44	0.971	0.383
Preoperative CSA (cm^2^)	0.41 ± 0.13	0.50 ± 0.19	0.70 ± 0.26^#^	10.808	0.000
Preoperative low back pain VAS (score)	5.86 ± 1.10	5.09 ± 0.77	4.48 ± 0.75^#^	16.764	0.000
Preoperative leg pain VAS (score)	5.28 ± 0.91	4.95 ± 1.12	4.00 ± 0.56^#^	20.747	0.000
Preoperative ODI (%)	57.07 ± 3.34	54.90 ± 4.40	47.27 ± 3.34^#^	59.193	0.000

^#^Indicates that the difference between Group 3 and Group 2, Group 3 and group 1 is statistically significant

DH, Disc height; LLA, Lumbar lordotic angle; CSA, cross-sectional area; VAS, visual analogue scale; ODI, Oswestry disability index

**Table 5:** Comparative analysis of pre- and post-operative efficacy in Group 1 patients

	Preoperative	Postoperative	Paired t value	*P*
Number	14	14		
low back pain VAS (score)	5.86 ± 1.10	3.93 ± 0.83	11.720	0.000
leg pain VAS (score)	5.29 ± 0.91	3.64 ± 1.01	9.706	0.000
ODI (%)	57.07 ± 3.34	51.00 ± 3.11	8.153	0.000

VAS, visual analogue scale; ODI, Oswestry disability index

**Table 6:** Comparative analysis of pre- and post-operative efficacy in Group 2 patients

	Preoperative	Postoperative	Paired t value	*P*
Number	21	21		
low back pain VAS (score)	5.10 ± 0.77	3.10 ± 0.44	11.832	0.000
leg pain VAS (score)	4.95 ± 1.12	2.71 ± 0.84	7.270	0.000
ODI (%)	54.90 ± 4.40	44.19 ± 3.79	8.977	0.000

VAS, visual analogue scale; ODI, Oswestry disability index

**Table 7:** Comparative analysis of pre- and post-operative efficacy in Group 3 patients

	Preoperative	Postoperative	Paired t value	*P*
Number	52	52		
low back pain VAS (score)	4.48 ± 0.75	2.98 ± 0.83	13.874	0.000
leg pain VAS (score)	4.00 ± 0.56	2.98 ± 0.83	8.875	0.000
ODI (%)	47.27 ± 3.34	37.62 ± 3.50	49.281	0.000

VAS, visual analogue scale; ODI, Oswestry disability index

The general clinical data of patients in Group 1 and Group 2 were compared as shown in Table 8. The results showed that there was no significant difference in Age, Sex, Symptom duration, BMI and Number of stenosis segments between Group 1 and Group 2. The comparison of postoperative clinical and radiographic data between Group 1 and Group 2 is shown in Table 9. Patients in Group 2 showed greater recovery of dural sac CSA than patients in Group 1, and the postoperative ODI score of patients in Group 2 was better than that of patients in Group 1. According to the difference of the midpoint position of the fusion cage relative to the intervertebral space, we divided the position of the fusion cage into three different positions: anterior, middle and posterior [17]. The position of the fusion cage in both groups was located in the anterior or middle part, and the number of use of the posterior fusion cage was 0. Specifically, the cage position (anterior: middle) of patients in Group 1 and Group 2 was (10: 4) and (2: 19), respectively, and the difference between the two groups was statistically significant.

**Table 8:** Comparison of demographic data between Group 1 and Group 2 Patients

	Group 1	Group 2	t/X^2^	*P*
Number	14	21		
Age (years)	65.50 ± 9.22	65.81 ± 11.29	0.085	0.933
Sex ratio (M: F)	6:8	8:13	0.079	0.778
Symptom duration (months)	44.21 ± 44.80	47.33 ± 78.95	0.134	0.894
Height (cm)	164.28 ± 5.94	165.19 ± 5.07	0.483	0.633
Weight (kg)	65.21 ± 7.85	66.02 ± 8.19	0.291	0.773
BMI (kg/m^2^)	24.10 ± 2.13	24.19 ± 2.81	0.099	0.922
Number of stenosis segments	1.86 ± 0.36	1.62 ± 0.50	1.635	0.112

**Table 9:** Comparison of postoperative clinical and radiographic data between Group 1 and Group 2 patients

	Group 1	Group 2	t/X^2^	*P*
Number	14	21		
Operation time (hours)	1.65 ± 0.46	1.76 ± 0.80	0.497	0.622
Blood loss (ml)	76.43 ± 29.25	63.33 ± 24.15	1.444	0.158
Postoperative DH (cm)	1.51 ± 0.18	1.64 ± 0.19	2.091	0.044
Pre- and post-operative DH difference (cm)	0.42 ± 0.18	0.53 ± 0.21	1.592	0.121
Postoperative LLA (°)	44.46 ± 8.48	43.16 ± 15.93	0.313	0.757
Pre- and post-operative LLA difference (°)	4.65 ± 8.17	−2.76 ± 10.73	2.191	0.036
Postoperative CSA (cm^2^)	0.52 ± 0.16	0.91 ± 0.38	4.138	0.000
Pre- and post-operative CSA difference (cm^2^)	0.11 ± 0.16	0.41 ± 0.34	3.386	0.002
Postoperative low back pain VAS (score)	3.93 ± 0.83	3.10 ± 0.44	3.456	0.003
Pre- and post-operative low back pain VAS difference	−1.93 ± 0.62	−2.00 ± 0.77	0.289	0.774
Postoperative leg pain VAS (score)	3.64 ± 1.01	2.71 ± 0.84	2.948	0.006
Pre- and post-operative leg pain VAS difference	−1.64 ± 0.63	−2.24 ± 1.41	1.694	0.101
Postoperative ODI (%)	51.00 ± 3.11	44.19 ± 3.79	5.577	0.000
Pre- and post-operative ODI difference	−6.07 ± 2.79	−10.71 ± 5.47	2.923	0.006
Cage position (anterior: middle)	10:4	2:19	14.287	0.000

DH, Disc height; LLA, Lumbar lordotic angle; CSA, cross-sectional area; VAS, visual analogue scale; ODI, Oswestry disability index

## Discussion

RNRs are a special phenomenon in patients with LSS, which is characterized by the winding, meandering and tortuous state of the cauda equina nerve root in the lumbar spinal canal [21–23]. Although many studies have been published on RNRs, the exact mechanism of RNRs is still unclear. The observation of the spatial distribution of redundant nerve roots and the degree of degeneration of nerve fibers in redundant nerve roots showed that there was a close causal relationship between RNRs and spinal stenosis. The pathogenesis of RNRs is considered to be related to nerve root compression caused by spinal canal stenosis, especially in LSS patients, which leads to mechanical compression of nerve roots. This squeeze limits the normal movement of the nerve root in the head–tail direction. When the stenosis is severe, the head end of the cauda equina nerve may not be able to move freely to the narrow tail end. In addition, repeated movement of the lumbar spine may gradually push the nerve root out of the spinal canal at the site of stenosis, and over time, the nerve root may become redundant and lengthen [22–25].

In this study, we found that patients with RNRs had more stenotic segments, smaller preoperative dural sac CSA, and may have more severe symptoms. The age, sex, course of disease and BMI of patients were not correlated with the occurrence of RNRs, which was statistically significant. In the study of Rousan et al. [26],contrary to the study of Papavero et al. [27],they did not find a significant statistical correlation between the diversity and degree of stenosis and the occurrence of RNRs. In addition, Yokoyama et al. [14] divided 33 LSS patients into redundant group and non-redundant group according to the MRI scan on the 7th day after operation. Hur et al. [28] conducted a retrospective analysis of 106 patients undergoing decompression surgery. The results showed that long course of disease and multi-segmental stenosis were related to RNRs, but the severity of symptoms was not related to redundancy. At the maximum stenosis level, the CSA of the dural sac in the redundant group was significantly reduced, which was consistent with our findings.

Although several studies have evaluated the occurrence of RNRs, the clinical efficacy of RNRs is still unclear. Our study observed that patients improved after OLIF combined with percutaneous internal fixation regardless of the presence or absence of RNRs. The postoperative VAS and ODI scores of the three groups were significantly improved compared with those before operation. Early studies have shown that there are differences in the efficacy of RNRs patients. For example, Suzuki et al. [22] conducted a comprehensive RNRs study in 1989, investigating myelography in 1256 patients with lumbar disease. Among 130 patients with severe LSS, 55 (42%) patients were found to have RNRs. In their study, 21 (91%) of 23 patients showed complete regression of RNRs by postoperative myelography, and the symptoms of all patients improved. Min et al. [15] retrospectively analyzed 68 patients who underwent laminectomy due to single-segment lumbar spinal stenosis and divided them into redundant and non-redundant groups. Among them, 23 patients (33%) had RNRs on preoperative MRI. The results showed that there was no statistical difference in Japanese Orthopedic Association (JOA) score, spinal canal diameter or recovery rate before or 1 year after surgery. Although there was no significant difference in surgical results between the redundant group and the non-redundant group, the surgical results of the non-redundant group were slightly better. These findings highlight the complexity and uncertainty of the therapeutic effect of RNRs on LSS patients, so the findings of this study are of great significance for further understanding the treatment options of RNRs patients.

Different from the previous surgical methods such as posterior lumbar interbody fusion for the treatment of patients with RNRs, OLIF has unique advantages in dealing with patients with severe RNRs. The risk of dural sac rupture may occur in the traditional posterior approach, especially in the presence of severe adhesion of dural sac. Xu et al. [29] noted in their study that in the application of posterior lumbar interbody fusion with total laminectomy for RNRs patients, adhesions around the cauda equina are frequently encountered. Consequently, meticulous and cautious technical approaches are essential when performing decompression of redundant nerve roots. However, OLIF uses an oblique lateral approach for indirect decompression, by inserting a larger cage to open the intervertebral space to restore the height of the intervertebral space and the height of the intervertebral foramen, while stretching the posterior longitudinal ligament and the ligamentum flavum, thereby effectively reducing spinal stenosis [30, 31]. The uniqueness of this approach is that it avoids the risk of dural sac rupture from the posterior approach, making it a favorable choice for patients with RNRs. The results of this study showed that the dural sac CSA and DH were significantly improved after indirect decompression by OLIF, which was statistically significant. Fujibayashi et al. [32] performed OLIF combined with percutaneous internal fixation on 28 patients with degenerative diseases (including lumbar spinal stenosis). The results showed that all patients had clinical improvement after operation. The dural sac CSA increased from 99.6 mm ^2^ before operation to 134.3 mm^2^ after operation. DH, segmental intervertebral disc angle and clinical results were significantly improved. The results of Limthongkul et al. [33] showed that the average CSA of dural sac increased by 50.8% after OLIF. Shimizu et al. [34] also found that after OLIF indirect decompression treatment, the dural sac CSA increased by 72% after 3 weeks of follow-up.

However, why do preoperative RNRs positive patients remain positive after OLIF combined with percutaneous internal fixation, and their imaging and clinical efficacy are worse than those of negative patients. This is the most concerned issue in this study. According to the difference of the midpoint position of the cage relative to the intervertebral space, we divided the position of the cage into three different positions: anterior, middle and posterior [18], so as to further compare the imaging findings and surgical effects of patients in Group 1 and Group 2 before and after operation. The position of the cages in both groups was located in the anterior or middle part, and the number of use of the posterior cages was 0. Specifically, the cage position (anterior: middle) of patients in Group 1 and Group 2 was (10:4) and (2:19), respectively. It is worth noting that the improvement of ODI score and dural sac CSA in Group 2 was significantly better than that in Group 1 after operation. Relevant studies indicate that the positioning of the fusion cage significantly influences the success and postoperative outcomes of OLIF procedures. Placing the fusion cage in the middle or posterior may offer a more effective indirect decompression effect [18, 35–37]. The mechanical compression theory of RNRs can be traced back to 1992, which was proposed by Suzuki et al. [23] In his theory, RNRs is caused by secondary spinal stenosis. In this study, we found that some patients with positive RNRs turned negative after operation, while some patients with positive RNRs still had cauda equina redundancy after operation. Based on the results of this study and previous literature reports [18, 35–37], we speculate that it may be because OLIF surgery can achieve indirect decompression effect to a certain extent, relieve nerve compression and restore nerve function. However, the different positions of the fusion cage will make the above decompression results different, that is, the middle position of fusion cage can better expand the dural sac CSA than the anterior position, which plays a very important role in promoting the negative conversion of RNRs. Nevertheless, there is one fact that still needs our attention. The fusion device placed in the front may better increase the lumbar lordosis, but the increase of CSA is very limited, the fusion device placed in the middle can well increase CSA, but the improvement of LLA is very limited.

## Limitation

This study has conducted a certain degree of research on the position of the fusion cage and the postoperative outcome and clinical efficacy of RNRs, but there are still some limitations. Firstly, the sample size is relatively small and the follow-up time is limited, which may restrict the universality and reliability of our research results. Secondly, although we did not observe a significant correlation between patient age, course of disease and RNRs in this study, we cannot ignore the potential impact of such factors on RNRs. Finally, variations in surgical techniques, diverse patient comorbidities, or alterations in postoperative care protocols may significantly influence the study's conclusions. In subsequent research endeavors, a more thorough exploration of these specific factors is warranted to comprehensively fathom their impact on the study's outcomes.

## Conclusions

In summary, irrespective of the presence or absence of RNRs, patients experienced improvement after undergoing OLIF combined with percutaneous internal fixation. Preoperative RNRs appear to be linked to multi-segmental lumbar spinal stenosis, a reduction in dural sac CSA, and symptom severity. Patients with negative postoperative RNRs demonstrated better treatment efficacy. Furthermore, the placement of the fusion cage appears to have a significant impact on postoperative efficacy and RNRs outcomes.

## Data Availability

The datasets used and/or analyzed during the current study are available from the corresponding author on reasonable request.

## Abbreviations

LSS: Lumbar spinal stenosis
LIF: Lumbar interbody fusion
OLIF: Oblique lumbar interbody fusion
RNRs: Redundant nerve roots
MRI: Magnetic resonance imaging
CSA: Cross-sectional area
DH: Disc height
LLA: Lumbar lordotic angle
VAS: Visual analog scale
ODI: Oswestry disability index
BMI: Body mass index
